# Culturally Tailored Dietary Interventions for Improving Glycaemic Control and Preventing Complications in South Asians with Type 2 Diabetes: Success and Future Implications

**DOI:** 10.3390/healthcare11081123

**Published:** 2023-04-13

**Authors:** Grace Farhat

**Affiliations:** Faculty of Health and Education, Manchester Metropolitan University, Manchester M15 6BG, UK; g.farhat@mmu.ac.uk

**Keywords:** type 2 diabetes management, glycaemic control, south Asian population, diet, physical activity

## Abstract

Glycaemic control is the basis of type 2 diabetes mellitus (T2DM) management and is crucial for preventing diabetes microvascular and macrovascular complications. The South Asian population is at higher risk of T2DM and resultant cardiovascular disease, peripheral vascular disease and death compared to Caucasians. Effective diabetes care has been deemed challenging in this population, but little is known about the usefulness of lifestyle interventions in improving glycaemic control and reducing complications. This narrative review aims to explore the efficacy of lifestyle interventions targeted to South Asians with T2DM in inducing clinically relevant improvements in HbA1c levels at such levels that reduce the risk of diabetes complications. A search of the literature using six databases (MEDLINE (EBSCOhost), PubMed, CINAHL, PsycINFO, Cochrane Central Register of Controlled Trials and Scopus) identified dietary-based, physical-activity-based and education-based interventions that aimed to manage T2DM in South Asians. Results showed that dietary and physical activity interventions (duration 3-12 months) have been effective in generating a clinically relevant decrease in HbA1c levels (≥0.5%) in South Asians with T2DM and could potentially assist in reducing diabetes complications. Education-based interventions produced small effects on glycaemic control. These outcomes support the development of comparable longer-term randomised clinical trials combining dietary and physical activity interventions with the aim to provide further evidence on specific interventions that can lower complications and ensure effective diabetes care in a high-risk population.

## 1. Introduction

Type 2 diabetes is increasing at alarming rates and has reached 422 million people worldwide [1]. Whilst it is primarily caused by excess body weight, T2DM is exacerbated by poverty and lack of access to adequate health care [2,3]. People living in low-income and medium-income countries account for 90% of the global diabetes cases [4]. T2DM generates significant healthcare costs, which are expected to reach USD 2.2 trillion by 2030 [5]. Uncontrolled diabetes is a major cause of cardiovascular disease, stroke, peripheral vascular disease (retinopathy, neuropathy and nephropathy), disability and death [6,7]. Effective diabetes care and glycaemic control in patients with T2DM are crucial to slow the progression of the condition and prevent complications [8].

Pharmacotherapy and lifestyle modification (adherence to a healthy diet, physical activity) are usually recommended to maintain glycaemic control [9]. Modest weight loss (5–7%) has been shown to improve blood glucose control and lessen the reliance on glucose-lowering medications [10]. However, many patients with T2DM face difficulties in adhering to a healthy lifestyle regimen and following nutrition recommendations [11,12]. According to NICE (National Institute of Care and Excellence) guidelines, the evidence-based recommendations for health and care in England, a decrease in HbA1c levels of at least 0.5% (5 mmol/mol) is effective in reducing complications in patients with T2DM [13]. Achieving an HbA1c target level of 6.5% (48 mmol/mol) (NICE guidelines) or 7% (53 mmol/mol) (American Diabetes Association) in patients with T2DM can reduce mortality as well as microvascular and macrovascular complications [13,14].

People of South Asian origin refers to people from India, Pakistan, Bangladesh, Nepal, Bhutan and the Maldives [15]. They have a higher prevalence of T2DM compared to Caucasians, and it develops at a younger age and a lower weight [16,17]. It is estimated that around 70 million South Asians have T2DM [18], and this number is expected to rise to 121 million in 2030 [19]. This elevated risk can be ascribed to a combination of biological factors (higher ectopic fat deposition, lower beta cell reserve, higher levels of inflammation rates) and lifestyle factors (diets high in refined carbohydrates and saturated fats, low levels of physical activity) [17,19,20]. South Asians have a poorer glycaemic control, which can partly explain the higher incidence of micro- and macrovascular complications compared to Europeans [21]. A lack of motivation, low adherence to lifestyle modification advice, lack of awareness of the urgency of T2DM, inadequate knowledge of nutritional content of foods and absence of family support are also potential contributors to the challenges of controlling diabetes in this population [22,23]. In a Nepalese study, only 20% of patients with T2DM had controlled diabetes [24]. A large observational study in India showed that just 4% of patients with T2DM followed dietary recommendations [25].

Programmes focusing on diabetes management have attracted significant interest, but little is known about what specific interventions are effective in generating clinically relevant changes in HbA1c levels and potentially achieving HbA1c targets. T2DM remission through a very low-calorie diet (~850 Kcal) [26] has gained wide recognition over the last few years and its feasibility has also been reported in the South Asian population [27,28]. However, these diets, which create a significant calorie deficit, may not be widely acceptable among South Asians and can result in a lack of adherence [29]. Additionally, remission trials have primarily included patients diagnosed with T2DM within the last 6 years [27,28]. It is therefore unclear whether remission is possible in those who have had T2DM for a longer duration. This review aims to look at the usefulness of lifestyle interventions targeted to South Asians in generating clinically relevant improvements in glycaemic control to reduce diabetes complications. It will help identify common elements of successful interventions that can shape future studies and recommendations to improve this population’s well-being and reduce the significant economic health burden of diabetes.

## 2. Materials and Methods

### Search Strategy

A literature search of MEDLINE (EBSCOhost), PubMed, CINAHL, PsycINFO, Cochrane Central Register of Controlled Trials and Scopus was carried out in February 2023 with no time restrictions, using the following keywords: (Asian OR Indian OR Pakistani OR Bangladeshi OR Sri Lanka OR Nepal OR Maldives OR Bhutan) AND (Type 2 diabetes). The search was limited to the English language and to full-text articles. Reference lists of included studies were also searched for eligible studies.

Studies were restricted to South Asian patients with T2DM. Lifestyle interventions (randomised controlled trials or quasi-experimental studies) aiming to improve diet and/or physical activity for diabetes management and with a duration of ≥3 months were included. There were no restrictions to country or study setting. The primary outcome was a change in HbA1c levels. Secondary outcomes included changes in other measures of glycaemic control (fasting blood glucose (FBG) and/or postprandial blood glucose) and changes in weight status (body weight or BMI). Exclusion criteria included studies involving non-South Asians with a duration of less than 3 months and/or investigating participants with no history of T2DM. We adopted the DCCT unit for HbA1c (%) in this review as we were unable to convert available data into mmol/mol.

## 3. Results

The initial search resulted in 14,685 articles. After removing duplicate records (n = 5186) and records that were marked ineligible by automated tools (n = 4782), 4717 records were screened, and 102 studies were assessed for eligibility. Twenty studies met the inclusion criteria.

### 3.1. Study Characteristics

The studies’ durations were between 3 and 12 months. The studies included Indians (n = 15), Nepalese (n = 2), Pakistanis (n = 1) and Bangladeshis (n = 1), and 1 study included patients from mixed South Asian sub-ethnicities (n = 1). The studies were held in India (n = 14), Nepal (n = 2), the USA (n = 2), Canada (n = 1) and the UK (n = 1). Lifestyle interventions included education-based interventions (including education elements on a healthy diet and physical activity) (n = 8), dietary-based interventions (including advice on specific diets) (n = 3) and physical-activity-based interventions (including physical activity regimens (n = 8), from which 2 studies involved yoga). Studies assessed HbA1c levels (n = 18), FBG (n = 10) and postprandial glucose levels (n = 4). Three studies were quasi-experimental as they did not include a control group. The study characteristics are presented in Table 1.

### 3.2. Effects of Lifestyle Interventions on Glycaemic Control

#### 3.2.1. Education-Based Interventions

Studies including an education element (delivered through SMS messages, phone apps, flashcards or face-to-face group sessions) did not typically report clinically relevant improvements in glycaemic control. Whilst four studies reported a statistically significant decrease in HbA1c levels [30,33,35,36], only one 3-month intervention reported an overall clinically relevant decrease in HbA1c levels (−0.65% (0.96), *p* = 0.01) with 40% of participants achieving an over 1% decrease in HbA1c [33]. Three studies reported a small but significant decrease in fasting blood glucose after the intervention [31,32,34] (Table 1).

#### 3.2.2. Dietary-Based Interventions

Studies including *a* diet plan with or without partial meal replacement (duration of 3–6 months) reported clinically relevant decreases in HbA1c levels (−0.94% (0.15), *p* = 0.04 [37]; −0.62% (0.65), *p* = 0.01 [38]; and −0.87%, *p* = 0.001 [39]) (Table 1).

#### 3.2.3. Exercise-Based Interventions

Studies including aerobic or resistance exercise (duration of 3–12 months) reported the most significant improvement in HbA1c levels [40,41,42,43,46,47,48] (Table 1). The duration of the studies or the type of exercise (aerobic versus resistance exercise) did not appear to affect outcomes. In studies assessing different markers of glycaemic control, the decrease in FBG and postprandial blood glucose were in line with the decrease in HbA1c levels [40,41,42,43]. Yoga, a widely acceptable activity among many South Asian cultures, led to a lesser yet clinically relevant decrease in HbA1c levels (−0.5% (1.49), *p* = 0.02) [48], as well as a significant decrease in FBG and postprandial glucose levels [47,48]. Nonetheless, there was a significant heterogeneity between interventions with notable differences in exercise regimen and intensity.

### 3.3. Effects of Lifestyle Interventions on Weight Status

Ten studies included a measure of weight status (weight (kg) and/or BMI (kg.m^2^). Overall, most education-based studies did not report a significant decrease in weight. However, dietary-based and education-based studies reported a significant decrease in weight, which was concomitant with the decrease in markers of glycaemic control. In dietary-based interventions, partial meal replacement led to the most significant effects on weight (−2.19 kg (3.14), *p* = 0.001) [37], while an aerobic exercise regimen of moderate intensity (150 min/week) for 6 months generated a mean decrease of 2.99 kg in weight (*p* = 0.001) [40]. Overall, the effectiveness of interventions did not appear to differ between studies either held in the South Asian region or in countries where South Asians have migrated.

## 4. Discussion

This narrative review aimed to evaluate the success of lifestyle interventions in inducing clinically significant improvements in glycaemic control at such levels that reduce micro- and macrovascular complications in South Asians with T2DM. With this population making up a quarter of the world’s population and having a high prevalence of diabetes [15], cost-effective interventions that improve the quality of life of South Asians and lessen the economic burden of the disease are urgently needed.

Dietary and physical activity interventions (duration of 3–12 months) appeared to generate a clinically relevant decrease in HbA1c levels (≥0.5%), which was accompanied by a significant weight loss, yet the short duration of the studies did not allow for an exploration of the impact on diabetes complications. Most physical activity interventions involving either aerobic or resistance training generated a mean decrease in HbA1c levels of ≥1% [40,41,43,44,45]. This level of reduction has been reported to be associated with a decrease in the risk of myocardial infarction (by 14%), microvascular complications (by 37%) and diabetes-related death (by 15%) [49]. Nevertheless, neither intervention type attained a mean difference in HbA1c levels below the target levels of 6.5 or 7%. While achieving target levels of HbA1c is recommended, it is indeed difficult for patients with T2DM to achieve near-normal HbA1c concentrations in real-life situations [49]. The intensification of lifestyle interventions would probably generate better outcomes but may be faced with reluctance and a lack of adherence in this population.

Studies involving 150 min of aerobic exercise/week for 6 months decreased HbA1c from 10.44% (2.24) to 8.45% (1.32) (*p =* 0.001) and improved blood pressure and lipid levels [43]. Similar interventions with longer durations may then prove to be feasible and effective in South Asians. Physical activity is particularly important in this population in view of the lower oxidative capacity and fatty acid metabolism and resultant higher insulin resistance compared to Europeans [50]. It has even been suggested that South Asians should consider carrying out 230 min of moderate exercise per week, which is above the guidelines of 150 min recommended for white Europeans [51]. Yoga, a highly acceptable activity in multiple South Asian groups, could be a viable alternative. The study of Arumugam et al. (2020) reported that the improvement of glycaemic control after 6 months of daily yoga was fairly similar to the improvement noted after an intensive lifestyle intervention [48].

Partial meal replacement interventions were reported to have beneficial effects on glycaemic control [37,38] and could present a viable option in diabetes management studies. Additionally, switching to low glycaemic index foods has been effective in reducing HbA1c levels [39] and could be better accepted when the substitutions included culturally acceptable (or familiar) foods that do not cause drastic changes to the traditional diet [23]. It is worth noting that it is unlikely that the weight loss achieved in the included studies equated to 5%, which is recommended as a clinically significant decrease in weight [52]. It has been previously suggested that improvements in glycaemic control can be effective in reducing fatty liver independent of weight loss, suggesting the importance of optimising blood glucose levels for managing diabetes regardless of weight change [53].

Patient education is crucial in improving disease management and reducing complications [33,41], and technology has played a significant role in improving adherence and motivation [34]. Nevertheless, this review suggests that education-based interventions generated overall small benefits on glycaemic control. This outcome is in line with a large UK primary care study, which failed to achieve significant changes in HbA1c following the provision of a culturally adapted programme to reduce cardiovascular risks in South Asians with T2DM [54]. Education may then be better included in physical activity and/or dietary interventions to improve outcomes rather than as a standalone intervention.

*Implications*: Current interventions have shown potential success in lowering markers of glycaemic control, yet randomised controlled trials with longer durations looking at the success of lifestyle interventions in reducing diabetes complications are now required. These may potentially target both diet and physical activity with the inclusion of an education element. Suggestions could include interventions that incorporate advice on a healthy diet to create a moderate calorie deficit (e.g., reduce saturated fats, switch to low glycaemic index foods, increase the consumption of fibre) together with 150 min of physical activity/week. It is crucial to understand the barriers to physical activity among South Asians, which are majorly of a cultural and/or religious nature [55], and tailor the intervention accordingly. For example, a workout DVD made in the Bengali language was offered to Bangladeshi women to promote physical activity at home [39]. Moreover, incorporating weekly peer support sessions has been reported to have beneficial effects on diabetes management [56].

To increase the chances of success, future interventions should primarily consider the substantial sub-ethnic diversity within the South Asian population relating to patients’ educational levels, language, cultural background and socioeconomic status [33,36]. Feedback from the community has been considered crucial in creating acceptable interventions that increase compliance and adherence [36], whilst understanding that perceptions from a particular community may not be extrapolated to another. For example, while a group of UK South Asians preferred one-to-one interventions [33], Canadian South Asians considered a peer support group to be beneficial [57]. Lastly, involving dietitians in the delivery of lifestyle interventions, when possible, may produce better outcomes, as previously stated [35].

*Strengths and Limitations*: This is the first review that provided an overview of the effectiveness of lifestyle interventions in generating clinically relevant changes in HbA1c levels that can reduce micro- and macrovascular complications. It was, however, limited by the significant heterogeneity between included studies, the poor methodological quality of several studies, the unblinded design and lack of control group, the lack of between-group analyses in some studies and the missing data (including outcomes and attrition rate). Most studies also did not report information on medication adherence. This, in addition to differences in baseline body weight and HbA1c levels between studies, may have affected the outcomes. Lastly, while HbA1c is the gold standard for assessing and monitoring diabetes [58], the inflammation-mediated association between postprandial glucose levels and cardiovascular complications [59] suggests the benefit of additionally assessing this glycaemic marker in future interventions.

## 5. Conclusions

Dietary and physical activity interventions in South Asians with T2DM are effective in inducing clinically significant improvements in glycaemic control and could potentially reduce diabetes complications, even without achieving the recommended HbA1c target levels. The development of long-term well-designed studies targeting diet and physical activity with input from community members is now required to support these findings. Combining education with dietary and physical activity interventions could present a viable solution to help reduce diabetes complications and improve the quality of life of people living with the condition.

## Figures and Tables

**Table 1:** Characteristics of the included studies.

Study Characteristics	Length (Months)	Type of Intervention	Intervention Group	Control Group	Outcome Measures	Preintervention	Postintervention	Change from Baseline
Gupta et al., 2020 [30] India Two-arm RCT Indians N = 81 (39 F; 42 M) Age: 50.1 (9.4) years BMI: 27.7 (4.8) kg/m^2^ HbA1c: 7.5–10% Attrition rate: 3.7%	3–4 months	Education-based	Video-based lifestyle education programme (phone app) including advice on healthy diet and physical activity	Dietary advice; 30 min of brisk walking (at least 5 times/week)	HbA1c (%)	Control: 8.39 (0.65) Intervention: 8.51 (0.74)	8.38 (1.37) 7.86 (1.04) *	0.01 (1.29) −0.65 (0.96) *
Gyawali et al., 2021 [31] Nepal Two-arm RCT Nepalese N = 244 (120 F; 92 M) Age: 25–65 years Attrition rate: 13.11%	12	Education-based	Education on healthy diet and body weight, counselling for health promotion and behaviour change (delivered by female community healthy volunteers)	Usual care	FBG (mmol/L) BMI (kg/m^2^)	Control: 8.5 (2.4) Intervention: 8.7 (2.4) Control: 27.12 (4.01) Intervention: 26.74 (4.00)	8.9 (3.1) 7.5 (2.0) * 27 (4.07) 26.31 (3.90)	0.4 (2.0) −1.2 (2.2) * −0.12 (1.26) −0.43 (1.55)
Kumar et al., 2018 [32] India Two-arm RCT Indians N = 786 (622 F; 164 M) Attrition rate: 10.79%	12	Education-based	Individualised SMS messages (twice/month) including advice on healthy weight and healthy diet	Usual care	FBG (mmol/L) BMI (kg/m^2^)	Control: 8.4 (3.5) Intervention: 9.1 (3.7) Control: 24.4 (3.9) Intervention: 24.3 (4.1)	8.3 (4.0) 8.5 (3.7) * 25.2 (4.1) * 24.7 (5.0)	−0.1 −0.6 * 0.8 * 0.4
Hawthorne et al., 1997 [33] UK Two-arm RCT Pakistanis N = 201 (107 F; 94 M) Attrition rate: 4.48%	6	Education-based	Culturally appropriate pictorial flashcards for health education	No intervention	HbA1c (%)	Control: 8.6 (2.17) Intervention: 8.4 (2.43)	8.64(1.99) 8.3 (2.31) ^a^	0.04 −0.1 *
Islam et al., 2018 [23] USA Two-arm RCT Bangladeshis N = 336 (135 F; 201 M) Age: 21–75 years HbA1c ≥ 6.5% Attrition rate: 13.4%	6	Education-based	Group-based culturally tailored educational sessions (5 sessions) and one-to-one visit (2 sessions) from a community health worker	One educational group session	HbA1c (%) Body weight (kg) BMI (kg/m^2^)	Control: 8.0 (1.5) Intervention: 7.8 (1.3) Control: 69.22 (10.48) Intervention: 68.45 (11.29) Control: 27.3 (4.4) Intervention: 26.8 (4.2)	8.0 (1.6) 7.6 (1.2) 68.58 (10.66) * 67.45 (11.29) * 27.0 (4.3) * 26.4 (4.3) *	0 (1.18) −0.2 (1.23) −0.64 (3.08) * −1 (2.68) * −0.3 (1.33) * −0.4 (1.18) *
Shetty et al., 2011 [34] India Two-arm RCT N = 215 HbA1c: 7–10%	12	Education-based	SMS (once every 3 days) including recommendations to follow advice on dietary modification, physical activity and medication use	Usual care	FBG (mmol/L) Postprandial blood glucose (mmol/L)	Control: N/A Intervention: 10.3 (3.2) Control: N/A Intervention: 14.6 (4.7)	N/A 9.2 (3.0) * N/A 12.2 (3.7) *	−1.1 * −2.4 *
Wilson et al., 2003 [35] USA Retrospective study (audit) Indians N = 7490 (4645 F; 2901 M) Age: 55.2 (13.3) years	12	Education-based	Clinical nutrition education delivered by a registered dietitian or non-registered dietitian	No clinical education	HbA1c (%)	Control: N/A Intervention: N/A	N/A N/A	0.06 (2.7) −0.09 (2.18) *
Tang et al., 2022 [36] Canada Quasi-experimental study South Asians N = 114 (112 F; 2 M) Age ≥ 21 years	3	Education-based	Group-based diabetes self-management education and support sessions co-led by a diabetes educator and a peer leader (6 sessions; 2 h each) and only by a peer leader (6 sessions; 2 h each)	No control group	HbA1c (%)	8.21 (1.59)	7.82 (1.63) *	−0.38 (0.97) *
Dharmalingam et al., 2022 [37] India Two-arm RCT Indians N = 171 (64 F; 107 M) Age: 18–65 years HbA1c: 6.5–10% BMI: 23–24 kg/m^2^ Attrition rate: 19.32%	3	Dietary-based	Diabetes-specific nutrition supplement (DSNS) (50 g/day)	Usual care	HbA1c (%) Body weight (kg)	Control: 7.88 (0.85) Intervention: 8.05 (0.82) Control: N/A Intervention: N/A	7.72 (1.15) 7.42 (0.68) * N/A N/A	−0.16 (0.98) −0.62 (0.65) * −0.22 (2.03) −2.19 (3.14) *
Mohan et al., 2019 [38] India Two-arm RCT Indian N = 120 (45 F; 75 M) Age: 30–65 years BMI 23–30 kg/m^2^ HbA1c: 7–10% Attrition rate: 23.33%	3	Dietary-based	Counselling on health diet and physical activity (1400 Kcal/day) with diabetes-specific nutrition supplement (DSNS)	Counselling on health diet and physical activity (1400 Kcal/day)	HbA1c (%) FBG (mmol/L) Postprandial blood glucose (mmol/L) Body weight (kg) BMI (kg/m^2^)	Control Intervention Control Intervention Control Intervention Control Intervention Control Intervention	N/A	−0.48 (0.14) −0.94 (0.15) * 0.1 (0.3) −1.0 (0.3) * −0.1 (0.6) −1.6 (0.6) * −0.28 (0.25) −0.34 (0.26) −0.13 (0.09) −0.14 (0.1)
Pavithran et al., 2020 [39] India Two-arm RCT Indians N = 40 (20 F; 20M) Age: 35–65 years HbA1c 7–10% BMI < 35 kg/m^2^ Attrition rate: 10%	6	Dietary-based	Diet plan (by a dietitian) including foods with low glycaemic index	Usual diet	HbA1c (%) Body weight (kg) BMI (kg/m^2^)	Control: 8.1 (0.98) Intervention: 8.28 (0.91) Control: 73.12 (8.76) Intervention: 67.91 (12.56) Control: 27.25 (2.72) Intervention: 26.81 (5.04)	8.28 (0.91) 7.41 (0.89) * 73.40 (9.03) 66.02 (11.05) * 27.32 (2.78) 26.06 (4.23)	0.18 −0.87 * 0.28 (1.48) −1.88 (2.85) * 0.07 (0.56) −0.75 (1.23) *
Goit et al., 2017 [40] Nepal Quasi-experimental study N = 41 Nepalese BMI 32.94 (1.37) kg/m^2^	6	Physical-activity-based	Moderate intensity aerobic exercise (3 times/week; 50 min each session (10 min warm up, 30 min of brisk walking and light running and 10 min of cool-down)	No control group	HbA1c (%) FBG (mmol/L) Body weight (kg) BMI (kg/m^2^)	10.44 (2.24) 9.1 (0.4) 87.80 (4.22) 32.94 (1.37)	8.45 (1.32) * 6.3 (0.5) * 84.90 (4.58) * 29.95 (1.23) *	−1.99 * −2.8 * −2.9 * −2.99 *
Hariharasudhan and Varunkumar, 2015 [41] India Two-arm RCT N = 80 (All M) Indians Age: 30–60 years	3	Physical-activity-based	Supervised resistance training (10 exercises for major muscle groups) with increased intensity after 4 weeks	Usual care	HbA1c (%) FBG (mmol/L)	Control: Intervention: Control: Intervention:	N/A N/A N/A N/A	−1.41 *^a^ −1.2 mmol *
Misra et al., 2008 [42] India Quasi-experimental study Indians N = 30 (8 F; 22 M) Age: 24–50 years BMI: 17.5–30 kg/m^2^	3	Physical-activity-based	Resistance training (6 muscle groups, 2 sets, 10 repetitions each)	No control group	HbA1c (%) FBG (mmol/L) BMI (kg/m^2^)	7.7 (0.5) 10.07 (2.0) 24.1 (3.9)	7.2 (0.3) * 7.4 (1.2) * 24.1 (3.7)	−0.54 (0.4) * −2.7 (2.2) * 0.1 (1.1)
Shenoy et al., 2009 [43] India Three-arm RCT Indians N = 30 (14 F; 16 M) Age: 40–70 years BMI ≥ 23–30 kg/m^2^ Attrition rate: 0%	4	Physical-activity-based	Resistance training arm (PRT) (such as biceps curls, rriceps curls, front lateral pull down, knee extension exercise) twice/week. Aerobic exercise arm (AE) (walk 3 times/week; 30 min each)	No intervention	HbA1c (%) FBG (mmol/L)	Control: 7.77 (0.9) PRT: 7.57 (1.4) AE: 8.11 (0.9) Control: 10.6 (3.3) PRT: 9.6 (2.0) AE: 10.1 (1.9)	7.56 (0.9) 5.74 (0.8) * 6.78 (1.3) * 8.9 (2.9) 5.8 (0.7) * 8.7 (3.0) *	−0.21 −1.83 * −1.33 * −1.7 −3.8 * −1.4 *
Seshadri et al., 2012 [44] India Two-arm controlled trial Indians N = 18 (All F) Age > 60 years BMI > 30 kg/m^2^	6	Physical-activity-based	Walking 5 min/hour for most walking hours (daily)	Standard advice on diet and physical activity	HbA1c (%) Weight (kg)	Control: 8.34 (0.91) Intervention: 8.76 (1.55) Control: 76.9 (10.4) Intervention: 74.1 (7.80)	9.34 (1.76) 7.43 (0.81) 77.24 (10.26) 71.7 (7.79) *	−1.0 −1.33 0.34 −2.4 *
Sridhar et al., 2010 [45] India Two-arm RCT Indians N = 105 (47 F; 58 M) Age 40–70 years	12	Physical-activity-based	Supervised aerobic exercise (cycling or treadmill); 3 sessions/week; 50 min each	No exercise intervention	HbA1c (%)	Control: 8.7 (0.32) Intervention: 8.5 (0.38)	9.84 (0.53) 7.44 (0.44) *	1.14 −1.06 *
Subramanian et al., 2016 [46] India Two-arm RCT Indians N = 100 Age: 30–60 years	3	Physical-activity-based	Exercise (such as physio ball, sitting postures) 3 times/week	Usual routine	HbA1c (%) BMI (kg/m^2^)	Control: 7.72 Intervention: 8 Control: 28.5 Intervention: 26.60	8.0 (0.28) 7.25 (1.25) * 29.0 25.0 *	−0.28 −0.75 * −0.5 −1.6 *
Amita et al., 2009 [47] India Two-arm RCT Indians N = 41 (11 F; 29 M) Age: 35–65 years	3	Physical-activity-based	Yoga-nidra (45 min daily)	No intervention	FBG (mmol/L) Postprandial blood glucose (mmol/L)	Control: N/A Intervention: 8.9 (0.7) Control: N/A Intervention: 13.9 (1.2)	N/A 7.7 (1.3) * N/A 13.2 (1.7) *	−1.2 * −0.7 *
Arumugan et al., 2020 [48] India Retrospective study (2-arm controlled study) Indians N-146 (94 F, 52 M) Age: 20–70 years BMI ≥ 23 kg/m^2^	6	Physical-activity-based	Yoga (60 min daily)	Standard care	HbA1c (%) FBG (mmol/L) Postprandial blood glucose (mmol/L) Body weight (kg) BMI (kg/m^2^)	Control: 7.75 (1.88) Intervention: 7.4 (1.30) Control: 6.4 (2.5) Intervention: 5.6 (1.4) Control: 11 (3.8) Intervention: 9.7 (2.6) Control: 65.82 (12.36) Intervention: 68.82 (27.18) Control: 26.16 (5.06) Intervention: 27.18 (4.07)	8.05 (1.94) 6.90 (1.29) * 7.0 (2.3) * 4.9 (0.6) * 11.8 (4.1) 8.3 (1.7) * 67.33 (11.82) * 65.90 (10.28) * 26.92 (4.33) * 26.02 (0.51) *	0.3 (2.99) −0.5 (1.49) * 0.6 (3.1) * −0.6 (1.3) * −1.4 (2.2) * 1.51 (6.07) * −2.90 (2.37) * 0.76 (2.67) * −1.14 (1.2) *

PRT: progressive aerobic training; AE: aerobic exercise; N/A: not available. Data are represented as mean (SD); only available **data** are presented. * Significant difference from baseline/significant mean difference from baseline *p* ≤ 0.05. ^a^ Adjusted difference was −0.34% (95% CI −0.81: 0.13), *p* < 0.05. Table only includes available data.

## Data Availability

Not applicable.
